# Measurements of Radio‐Frequency Electromagnetic Field Levels in EXPO2025 Osaka, Kansai, Japan

**DOI:** 10.1002/bem.70062

**Published:** 2026-06-26

**Authors:** Teruo Onishi, Kazuhiro Tobita, Miwa Ikuyo, Kaoru Esaki, Masao Taki

**Affiliations:** ^1^ Electromagnetic Compatibility Laboratory National Institute of Information and Communications Technology Koganei Tokyo Japan

**Keywords:** 5G, exposure monitoring, large‐scale event, mobile phone systems, RF‐EMF level

## Abstract

In this study, we investigated radio‑frequency electromagnetic field (RF‑EMF) levels at the Expo 2025 Osaka, Kansai, Japan, a large‑scale international event that attracted approximately 26 million visitors during the event period. Spot and portable measurements were conducted for broadcast, 4G, 5G, and ISM bands in both indoor and outdoor environments. The observed E‑field strengths remained below the reference levels specified in the Japanese Radio Radiation Protection Guidelines, with the maximum E‐field strength corresponding to 0.4% of the guideline limits. The RF‑EMF levels measured at the venue were comparable to those measured in typical urban areas and consistent with findings from similar studies conducted overseas. These results confirm that the level of RF‑EMF exposure during high‑density public events in Japan remains below regulatory limits and exhibits behavior trends similar to those observed in ordinary urban environments.

## Summary

- RF‐EMF measurements using different methods at the large‐scale international event, EXPO2025.
- These results confirm that the level of RF‑EMF exposure during high‑density public events remains below the guidelines.

## 1 Introduction

To clarify the levels of radio‑frequency electromagnetic fields (RF‑EMFs) encountered in daily life, we conducted extensive measurements across various locations in Japan, including indoor and outdoor environments in both urban and suburban areas (Ikuyo et al. 2023; Liu et al. 2024; Onishi et al. 2021, 2023; Tobita et al. 2023). All measured values were confirmed to fall within the limits specified by the Japanese radio radiation protection guidelines (MIC 2018). In addition, we have carried out large‑scale nationwide measurements using car‑mounted measurement systems and made these results publicly available (NICT 2025).

RF‑EMF levels are expected to increase in environments where large numbers of people gather, such as major public events, and several studies on investigating such settings were previously reported (Gonzalez‐Rubio and Najera 2025; Vecsei et al. 2022; Villaescusa‐Tebar and Garcia‐Pardo 2025); however, there have been no reports on such events in Japan. In this paper, we report on RF‑EMF measurements conducted during Expo 2025 Osaka, Kansai, Japan (Expo 2025). The Expo was held from April 13 to October 13, 2025, on Yumeshima Island in Osaka City, attracting approximately 26 million visitors and the participation of more than 150 countries and international organizations. Under the theme “Designing Future Society for Our Lives,” the Expo served as a global platform for collaboration, showcasing innovations and cultural exchanges addressing challenges related to health, the environment, and emerging technologies. It contributed to progress toward the United Nations Sustainable Development Goals (SDGs) and advanced Japan's vision of Society 5.0 (CAO 2015), and featured notable elements such as the Grand Ring, diverse national pavilions, and a Virtual Expo offering immersive digital experiences.

Measurements were conducted on June 1 and 2, 2025, when the number of visitors was approximately 150,000 per day. The measured wireless systems included FM broadcasting (FM), digital television (TV), mobile phone systems including fifth‑generation (5G) and equipment operating in the Industrial, Scientific, and Medical (ISM) frequency bands.

## 2 Materials and Methods

**Figure 1:**
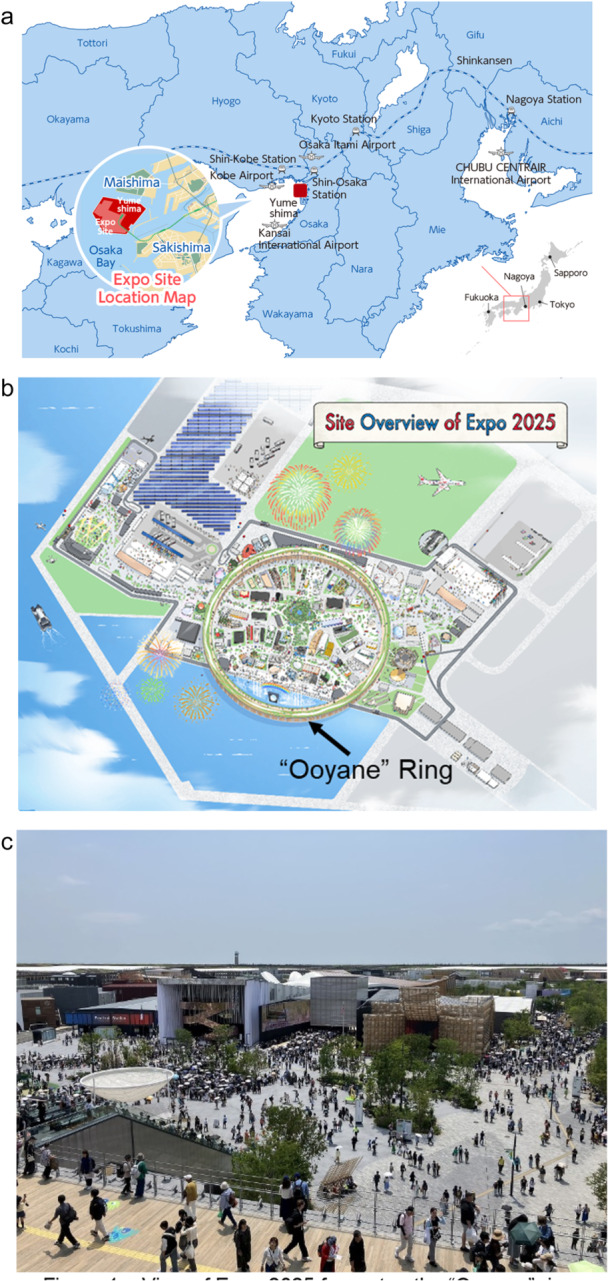
(a) Location of the Expo 2025 site in Kansai region, Japan. (Expo 2025). (b) Expo 2025 site overview (Expo 2025). (c) View of Expo 2025 from atop the “Ooyane” ring.

Measurements of electric fields (E‐fields) were carried out using spot and portable measurement methods on June 1 and 2, 2025 within the Expo site with an area of about 1.5 km^2^ (Figure 1). The measured frequency bands are allocated to FM, TV, mobile network of cellular systems, and ISM, whose frequency bands are shown in the Appendix. Since information on the mobile phone base stations is not disclosed in Japan, we visually checked the number of mobile phone base station antennas, which was 27 within the site.

Spot measurements were carried out in the exhibition hall (called Wasse) from 10:00 to 19:00 and in the parking lot from 9:00 to 22:00. An indoor measurement location was selected near an exhibit area where Beyond 5G (B5G) systems were demonstrated. This area showcased potential future societal and lifestyle changes enabled by next‐generation 5G technologies through video presentations, interactive experiences, and exhibits. The measurement equipment was installed behind the exhibit area, separated by a thin wooden partition so as not to disturb visitors. For the car‐based measurements, locations were chosen in proximity to the venue and within line of sight of the base station antenna.

**Figure 2:**
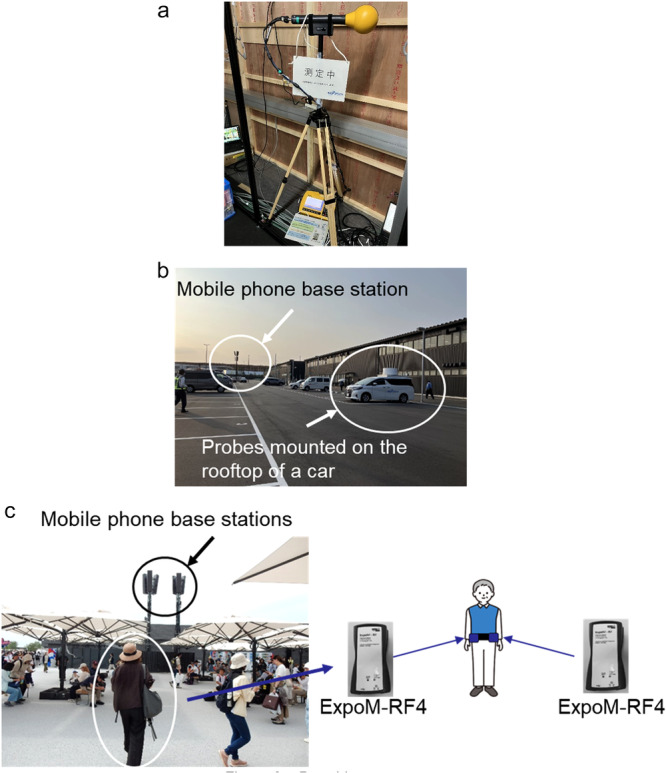
(a) Indoor spot measurement. (b) Oudoor spot measurement. (c) Portable measurement.

In indoor measurements, a spectrum analyzer (SRM‐3006, Narda S.T.S. GmbH, Germany) with a three‐axis isotropic E‐field probe (3502/01, Narda S.T.S; 420 MHz‐6 GHz) was used. This measuring instrument enables frequency selective measurement. The height of the probe was set at 1.5 m and measurements in the mobile phone base station downlink and ISM bands were mainly performed with a resolution band width (RBW) of 1 MHz (Figure 2a). Note that the results for the TDD band also include the uplink since the TDD band cannot be distinguished between the downlink and the uplink in this measurement. Measurements were performed continuously with a sweep time of about 2 s. For the outdoor measurement, two probes (Narda S.T.S. GmbH, Germany) for a low‐frequency (3501/03; 27 MHz‐3 GHz) and high‐frequency (3502/01) ranges were mounted on top of a car in the parking lot to measure the field strengths in the FM, TV, the mobile phone base station downlink, and ISM bands (Onishi et al. 2023). The probes were at a height of about 2.2 m from the ground. The E‐field strengths detected by the probes were recorded through each spectrum analyzer (Narda S.T.S. GmbH, Germany) installed in the passenger cabin. Although the distance of the probes from the Expo site was roughly 340 m, it was about 80 m from the nearest base station within the line of sight (Figure 2b). The RBW for FM and TV was also set at 200 kHz. In order to make the sweep times of the two probes roughly the same, 76 to 95 MHz and 700 to 2.7 GHz were measured for the probe (3501/03), and 470 to 710 MHz and 3.4 to 5.8 GHz were measured for the probe (3502/01). The time for measurement and recording for each probe was about 6 s.

For the portable measurement, two ExpoM‐RF4s (Fields at works GmbH, Switzerland) were carried around the waist of a measurer on the left and right sides. The frequency range supported by the instrument is 50 to 6 GHz. This measuring instrument is equipped with a three axis isotropic antenna and sequentially measures the set various preset frequency bands (see Appendix) with bandpass filter bandwidth (35, 75, 100 MHz). These three filters are combined to cover the desired frequency bands. This instrument records the E‐field strength within the filter bandwidth every 10 s. The measurement was conducted by walking within the site between 10:00 and 16:00 (Figure 2c). Unlike the spot measurement, the portable measurement can grasp the actual RF‐EMF levels by walking within the densely populated environment.

All measured data below the predetermined threshold (noise floor) are excluded. The remaining data were then calculated by the square root of the sum of the squares within dedicated frequency bands at each recording time (Onishi et al. 2023; Ikuyo et al. 2025). The data are averaged over 6 min.

## 3 Results

### 3.1 Spot Measurement

**Figure 3:**
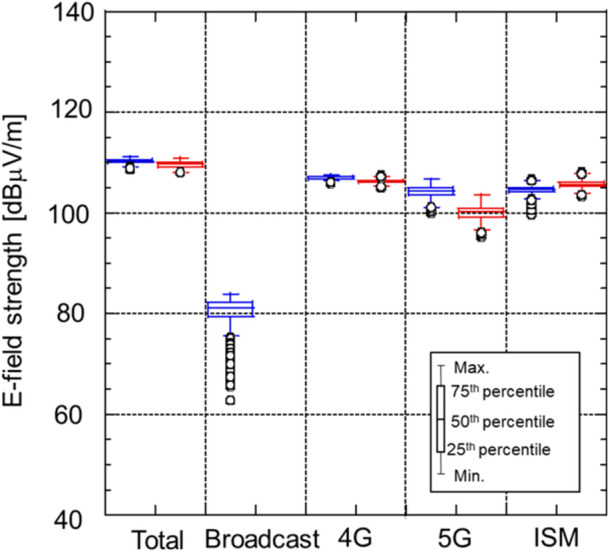
Box‐and‐whisker plot of E‐field by indoor spot measurement (blue; 1 June, red; 2 June).

**Figure 4:**
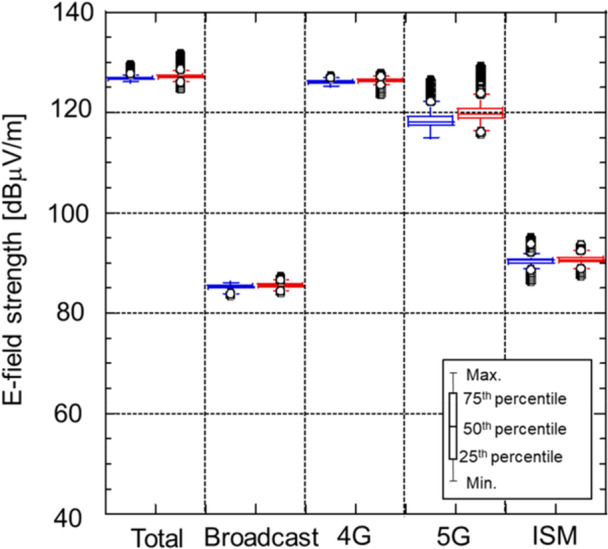
Box‐and‐whisker plot of E‐field by outdoor spot measurement (blue; 1 June, red; 2 June).

Figures 3 and 4 present box‑and‑whisker plots of the 6‑min‐averaged E‑field strength [dBµV/m] of broadcast (FM and TV), 4G, 5G, ISM, and the total across all measured bands for indoor and outdoor spot measurements, respectively, over the 2 days. Total represents the root‑sum‑square value across all measured frequency bands. The circles indicate outliers, defined as values greater than Q3 + 1.5 IQR or less than Q1 − 1.5 IQR, where IQR denotes the interquartile range. Overall, variations in E‑field strength for each system are small and this trend is consistent across the two measurement days. For indoor measurements, the E‑field strengths of all systems, except broadcast, fall within a similar level, approximately 100–110 dBµV/m. It should be noted that the E‑field strength of Broadcast (TV) measured on the second day was below the predetermined threshold. Most of the data points below the thresholds originate from indoor TV measurements.

For outdoor measurements, the E‑field strengths of 4G and 5G are definitely higher than those of broadcast and ISM. A comparison between indoor and outdoor environments shows that outdoor E‑field strengths for both 4G and 5G are approximately 20 dB higher than those observed indoors. Conversely, the outdoor ISM E‑field strength is lower than the indoor one, which can be attributed to the placement of Wi‑LAN access points.

### 3.2 Portable Measurement

**Figure 5:**
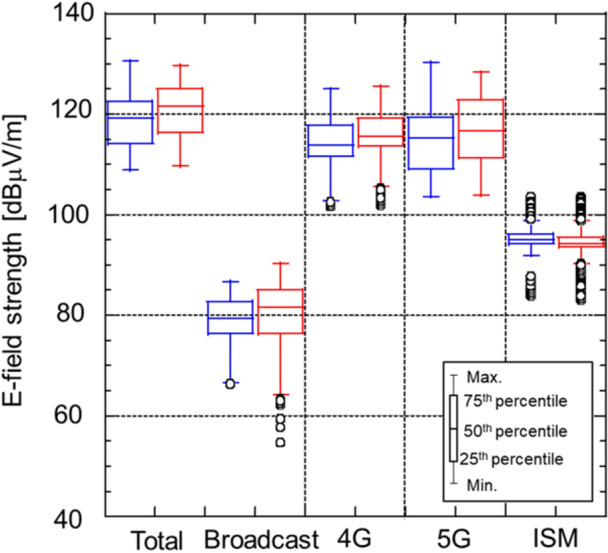
Box‐and‐whisker plot of E‐field by portable measurement (1 June, blue; left, red; right).

The results of the portable measurements conducted on the first day are shown in Figure 5. The same general trend as the outdoor spot measurements is observed. However, the variability observed in the portable measurements is larger, as the measurer moved through different locations and among large crowds (Figures 1c and 2c).

When comparing the left‑ and right‑side device positions, statistically significant differences were observed (*p* < 0.001); however, the degrees of the effect (Cohen's *d*) for all systems were small. Possible explanation for the difference is the effect of different tolerances on the measuring equipment. Another consideration is likely attributable to random variations in the relative distance and orientation with respect to the mobile phone base station during walking, resulting in a reduced difference between the left and right sides. Similar results were obtained on the second day, although the corresponding figure is omitted here.

## 4 Discussions

Even during the large‑scale event, the maximum E‐field strength observed in our measurements corresponded to 0.4% (−24 dB) of the reference level prescribed in the Japanese Radio Radiation Protection Guidelines (MIC 2018), which is similar to the ICNIRP guidelines (ICNIRP 2020). Villaescusa‐Tebar and Garcia‐Pardo (2025) reported a similarly low value of 0.31% (−25 dB) relative to the ICNIRP reference level, indicating that the levels measured in our study are comparable to those reported internationally.

The nominal measurement uncertainty of the SRM‐3006 is frequency‐dependent. For example, when the 3502/01 probe is used, the uncertainty ranges from +2 dB to −2.6 dB in the frequency band of 2–4 GHz. In contrast, the measurement uncertainty of the ExpoM‐RF4 is reported to be 4.5 dB.

In addition to the measurement instruments, several factors may influence the measurement results. For indoor and outdoor spot measurements, potential sources of uncertainty include nearby wooden partitions and structures such as vehicle bodies and radome, respectively. For portable measurements, human body shielding is considered a relevant factor.

According to a NIST report, the attenuation caused by a wooden wall is approximately 1–2 dB (NIST Construction Automation Program 1997). Although a wooden partition was present at the measurement site, it did not reach the ceiling, leaving the upper portion open. Therefore, its overall influence on the measurements is considered to be limited.

Regarding the influence of a vehicle body, previous work (Onishi et al. 2023) reported evaluations conducted in a large anechoic chamber capable of accommodating a vehicle. The results indicated that the difference between the measured and theoretical electric field strengths was within ±2.5 dB.

For portable measurements, it has been reported that carrying two devices, one on each side of the body, and averaging the measured values can reduce the uncertainty caused by human body shielding (Bolte et al. 2011). In the present study, although statistically significant differences were observed, the variation between the left and right sides was relatively small. Therefore, the influence of the human body on the statistical analysis is considered marginal.

Even when these uncertainties are taken into account, the measured values are confirmed to be below the reference levels.

We have previously conducted RF‑EMF measurements at numerous locations in Japan using various measurement methods. In our past results, the highest E‑field strength obtained in outdoor spot measurements was observed near a major train station at the center of Tokyo, and it reached 130.7 dBµV/m (Onishi et al. 2025). In comparison, the maximum median value in the present study, as shown in Figure 4 for the present study is 131.6 dBµV/m. The two results are about the same level since the locations have in common that they are environments in which a lot of people gather. In such a place, many mobile phone base stations are often located nearby.

For portable measurements conducted around the same major train station, the median E‑field strengths over 2 days were approximately 117.4 dBµV/m. In contrast, the maximum of median values in Figure 5 is 121.7 dBµV/m, which is 4.3 dB higher. This may be because, in the situation of past portable measurements, the shopping street was slightly farther from the train station and the crowd density was not that high (Ikuyo et al. 2025).

Regarding 5G, we also previously reported measurements of 5G FR1 signals across 51 locations in Tokyo, conducted both with and without data downloads to a smartphone (Tobita et al. 2024). The corresponding maximum E‑field strengths were 106.0 dBµV/m without data transfer and 123 dBµV/m with data transfer. In the present study, measurements were conducted only without data transfer condition; however, the median E‑field strength was approximately 120 dBµV/m. This is different from the previously observed trend and is at the same level as the field strength with data download. The reason is considered to be caused by differences in the transmission power of mobile phone base stations, installation height, distance to the measurement site and so on. It is noted that mobile phone base stations in the Expo site include antennas among different mobile network operators. Recently, the sharing of infrastructure has progressed, but in the past, each operator in Japan installed their own base station antennas at different locations.

## 5 Conclusions

We presented RF‑EMF measurements conducted during Expo 2025 Osaka, Kansai, Japan, one of the largest public events held in the world. The results of both spot and portable measurements demonstrated that the observed E‑field strengths across broadcast, 4G, 5G, and ISM bands remained below the reference levels prescribed in the Japanese Radio Radiation Protection Guidelines. Even under conditions where large numbers of visitors were present, the maximum measured E‐field strength corresponded to 0.4% of the guideline limit. Comparisons with our previous nationwide and metropolitan measurements showed that the RF‑EMF levels measured at the Expo venue were within the range of typical urban outdoor environments, although slightly higher than those obtained from portable measurements around a major train station.

Overall, the results confirmed that RF‑EMF exposure levels at large public events in Japan remain below guideline values and exhibit characteristics consistent with those measured in typical urban environments. These findings contribute to the growing body of empirical evidence supporting the safety and stability of RF‑EMF levels in real‑world settings, including environments with a high population density.

## Ethics Statement

The authors have nothing to report.

## Conflicts of Interest

The authors declare no conflicts of interest.

## Data Availability

The data that support the findings of this study are available from the corresponding author upon reasonable request.

## 1

Table A1.

**Table A1:** Wireless systems and their frequency bands.

System	Band name	Frequency range [MHz]	Spot indoor	Spot outdoor	Portable
FM		76–95		•	•
TV		470–710	•	•	•
Mobile network of cellular system (4G)	700 MHz UL	718–748			•
	700 MHz DL	773–803	•	•	•
	800 MHz UL	815–845			•
	800 MHz DL	860–890	•	•	•
	900 MHz UL	900‐915			•
	900 MHz DL	945–960	•	•	•
	1500 MHz UL	1427.9–1462.9			•
	1500 MHz DL	1475.9–1510.9	•	•	•
	1700 MHz UL	1710–1785			•
	1700 MHz DL	1805–1880	•	•	•
DECT etc.	1900 MHz	1884–1920	•	•	•
Mobile network of cellular system (4G)	2000 MHz UL	1920–1980			•
	2000 MHz DL	2110–2170	•	•	•
Mobile network of cellular system (5G)	2300 MHz TDD	2330–2370	•	•	•
Wi‐LAN etc.	2400 MHz ISM	2400–2497	•	•	•
BWA	2500 MHz	2545–2645	•	•	•
Mobile network of cellular system (4G)	3500 MHz	3400–3600	•	•	•
Mobile network of cellular system (5G)	3700 MHz (FR1)	3600–4100	•	•	•
	4500 MHz (FR1)	4500–4600	•	•	•
Mobile network of cellular system (L5G)	4600 MHz	4600–4900	•	•	•
Wi‐LAN etc.	5200 MHz ISM	5150‐5250	•	•	•
	5300 MHz ISM	5250–5350	•	•	•
	5600 MHz ISM	5470–5730	•	•	•
